# Assessing the relative efficacy of different agents based on the outcomes from meta-analyses

**DOI:** 10.1111/j.1742-1241.2007.01594.x

**Published:** 2008-01

**Authors:** S Boonen, D Vanderschueren, P Haentjens

**Affiliations:** 1 Bone Research Unit, Leuven University Department of Experimental Medicine, Katholike Universiteit Leuven Leuven, Belgium; 2 Department of Orthopaedics and Traumatology, Vr'Je Universiteit Brussel Brussels, Belgium

*Dear Editor*,

referring to the publication of Liberman et al. (1) entitled ‘Hip and non-spine fracture risk reductions differ among antiresorptive agents: evidence from randomised controlled trials’, we question the authors’ conclusion that ‘Results from meta-analyses of fracture risk… suggest that ALN may be more effective than certain other antiresorptive agents in the treatment with osteoporosis’, based on their meta-analysis outcomes that alendronate (ALN) reduces the risk of hip and non-vertebral fracture by 45–55%, hormone therapy (HT) by 25–36% and risedronate (RIS) by 26–27%.

If the article's purpose was to summarise the effects of the currently approved antiresorptive therapies (page 1395, 1st paragraph), only the approved doses for each therapy should be included in the analysis. As the approved dose for ALN is 5 mg/day for prevention and 10 mg/day for treatment of osteoporosis, patients on ALN 20–40 mg/day dose should be excluded from the analyses. The meta-analysis for ALN included studies in which more than 50% of the ALN patients were treated with 20–40 mg/day dose (2,3). It does not appear that the authors addressed the scientific rationale for such an inclusion and its impact in the publication. The discussion on dose selection becomes particularly important when the authors suggested in the same article that the pooled effect size including the FIT trial was smaller because the patients used lower dose (5 mg/day) in the first 2 years (P1395, last paragraph).

The inclusion criteria of the clinical trials for the meta-analysis were inconsistent for the different therapies. As a result the treatment benefit the risedronate was underestimated. Non-osteoporotic patients were excluded from the meta-analysis for the ALN ≥ 5 mg/day and ≥ 10 mg/day doses, but in the RIS trials, patients who were over 80 years of age with risk factor(s) for fall without confirmed osteoporosis were included in the analyses. Note that patients who were over the age of 80 with difficulty of standing from a chair may not necessarily be osteoporotic (4). As neither ALN nor RIS demonstrated a significant treatment effect in patients without osteoporosis in the FIT or HIP trials, by including non-osteoporotic patients in the RIS analysis, the analysis is likely to underestimate the treatment effect of risedronate. The outcome of the sensitivity analysis (Table 3 of the article) suggested that it underestimated the risk ratio reduction hip fracture for RIS by 10% when including the non-confirmed osteoporotic group.

We believe that the authors may have simplified their outcomes and interpretation with their rationale that ‘Meta-analysis provides a more precise estimate than individual trials when the results are consistent across pooled trials’ and ‘This pooled analysis including FIT (≥ 5 mg/day) is not statistically justified (because of the significant interaction of treatment with dose)’. As pointed out by many authors in the literature, meta-analysis often ‘mixes apples with oranges’, the outcomes of the meta-analyses are not always reliable (5–7). It has been found that in many cases the outcomes of the meta-analyses later contradicted the outcomes of larger clinical trials (8). Further, the reasons for the inclusion/exclusion of a trial should be based on the clinical diversity of the trials not the results of the trial. ‘Decisions concerning what should and should not be combined are inevitably subjective, and are not amenable to statistical solutions but require discussion and clinical judgment’ (9).

We also note that different statistical methods were used for different therapies. While the majority of the outcomes were directly taken from Cranney et al. (10), which used a χ^2^ procedure, Poisson regression methods were used to update the meta-analyses for the therapies with new data. As a result, different statistical methods were used for different therapies. The rationale for using a different method was not disclosed in the publication and its impact was not explored for all agents.

Most importantly, as pointed out by Simon Richard, in Meta-Analysis in Medicine and Health Policy (11), the relative efficacy of different agents should not be compared based on the outcomes from meta-analyses. ‘Such indirect comparisons are usually not sufficient, because of differences among the studies with regard to numerous factors such as patients-selection criteria’ (11). Greater hip fracture risk ratio reduction was observed in the ‘fracture arm’ relative to the ‘non-fracture arm’ in both ALN FIT and RIS HIP trials. This suggests a significant impact of patient population on treatment efficacy. As patient selection criteria for ALN, HT and RIS trials were different, treatment comparisons based on the outcomes of individual meta-analyses may not have been appropriate in this publication.

Other differences between the ALN, HT and RIS trials included the inconsistent use of concomitant calcium and/or vitamin D, the differences in methodology in fracture assessment and classification, trial duration, and the rate of the loss to follow-up – all of which could contribute to the difference in the observed risk ratio reduction, if the difference exists at all. Note that no statistical procedure was conducted to substantiate any statistical significant difference in efficacy between therapies.

In conclusion, this meta-analysis has major limitations and therefore its conclusions may be misleading. The treatment group/dose from each trial was not selected to reflect the objective. The eligibility criteria for the inclusion of studies were not clearly defined which resulted in an inconsistency in trial selection for different agents. Different statistical methods were used to analyse different therapies and the basis to draw the conclusions was flawed.

We are concerned that this meta-analysis may not have been performed with the appropriate level of scientific rigour and that guidelines for systematic reviews (9) were not consistently followed.

Faulted on two grounds, the basic premise of meta-analysis vs. head-to-head clinical trials and the choice of study and treatment dose, the outcomes of any meta-analyses should be reviewed critically. In the present case, we believe that this meta-analysis was conducted without the appropriate level of scientific rigour or precision and the conclusions may not be supported by its results.
